# Ecological risk assessment of metal pollution in the surface sediments of delta region, Egypt

**DOI:** 10.1007/s10661-024-12481-w

**Published:** 2024-03-11

**Authors:** Walaa M. Thabet, Abeer A. Moneer, Ola Abdelwahab, Hoda H. H. Ahdy, Mohamed Khedawy, Nashwa A. Shabaan

**Affiliations:** 1 Marine Pollution Lab, Marine Environment Division, National Institute of Oceanography and Fisheries (NIOF), Cairo, Egypt; 2 Oceanography Department, Faculty of Science, Alexandria University, Alexandria, Egypt

**Keywords:** Distribution, Pollution indices, Sediment quality, Mediterranean Sea coast, Essential and toxic metals

## Abstract

The surface sediments from eleven sectors perpendicular to the Egyptian Mediterranean Sea coast, along the delta region, have been assessed. These sectors cover areas of Eastern Harbour, Abu Qir Bay, Rosetta, Abo Khashaba, Burullus, Baltim, Damietta, and Manzala. The assessment process is based on determining the eight metals’ (Fe, Cu, Zn, Mn, Ni, Co, Pb, and Cd) content, followed by applying different pollution and ecological risk indices such as enrichment factor (EF), geoaccumulation factor (*I*_geo_), contamination factor (CF), Pollution Load Index (PLI), degree of contamination (*C*_deg._), Nemerow Integrated Pollution Index (NIPI), and Potential Ecological Risk Index (PERI). The results indicate the average concentrations of metals (μg/g) were Fe (18,000), Mn (213), Zn (120), Ni (26), Co (13), Cu (12), Pb (10), and Cd (2). The single-pollution indices reveal that most metals were depleted to metal levels, the sediments were moderately polluted with Cd and unpolluted with other metals, and the CF values confirmed that the sediments were highly polluted with Cd and low contaminated with the rest of the metals. According to the integrated pollution indices, the Eastern Harbour, Damietta, Manzalah, and Bardawil (eastern Inlet) are the most polluted sectors relative to the rest, subjected to various point sources of pollution. Drainage management system is recommended to enhance water conservation and improve fish stocks.

**Supplementary Information:**

The online version contains supplementary material available at 10.1007/s10661-024-12481-w.

## Introduction

Anthropogenic activities have significantly impacted ecosystems in recent times, notably in aquatic environments (Fayanto et al., 2019). The rapid expansion of economic development and population growth has led to substantial pollutant release into marine ecosystems (Larsen et al., 2023). Ship traffic, mining, oil extraction, sewage discharge, ship repair, and ship repainting are the main human threats to marine ecosystems (Alharbi et al., 2023). Sediment acts as the primary source of metals in aquatic environments, with its quality serving as a potential indicator of water contamination levels (Shaaban, Shreadah, et al., 2021; Zahra et al., 2014). Acting as both a temporary sink and source of metals in aquatic environments (Ali et al., 2019; Fernandes and Nayak, 2012), sediments play a crucial role. The exceeding of limited metal values in water resources poses a severe environmental concern (Tepe et al., 2022; Thabet et al., 2023). Metals are considered hazardous contaminants due to their environmental persistence, biomagnifications, and cumulative behavior as well as their potential adverse effects (Madruga et al., 2014; Soliman et al., 2022; Ustaoğlu et al., 2020). Metal pollution in aquatic environments is caused by a variety of sources, including agricultural runoff, mining and industrial effluents, and domestic sewage (Ali et al., 2019; Nour et al., 2022; Thabet et al., 2020). These metals are distributed across the water, sediments, suspended solids, and biota within aquatic environments, potentially altering ecology and posing health risks to both humans and aquatic organisms (Shaaban, El-Rayis, & Aboeleneen, 2021; Shaaban, Tawfik, et al., 2021; Yunus et al., 2020). Numerous diseases, such as cancer, liver disease, nausea and vomiting, restrictive lung diseases, hypertension, kidney failure, abdominal pain, headaches, and nerve damage, have been linked to the chronic daily intake of high concentrations of some metals in the human system (Al-Kahtany, Nour, El-Sorogy, & Alharbi, 2023). Evaluations of the health hazards associated with metals have been carried out in numerous regions of the entire globe especially touristic and agricultural aquatic regions, with direct human contacts, which have been the focus of in-depth environmental research on coastal sediments, seawater, and benthic communities as well as increased anthropogenic influence (Alharbi et al., 2023; Al-Kahtany, Nour, Giacobbe, et al., 2023; Nour et al., 2022).

Egyptian Mediterranean coastal line extends about 970 km from Rafah in the east to Salloum in the west, with four natural lagoons, one artificial lake, and one natural lake extending from the Northern Sinai to Alexandria (El-Rayis et al., 2012). The Egyptian Nile Delta coastal line extends 240 km from Alexandria in the west to Port Said in the east (Mandour et al., 2021). The studied area occupies about a third of the Egyptian Mediterranean coastal line, which includes Eastern Harbour, the delta coastal area (Abu Qir, Rosetta, Abu Khashaba, Burullus Lagoon, Baltim, Gamasa, Damietta, and Manzalah Lagoon), in addition to Bardawil Lagoon perpendicular to the Egyptian Mediterranean Sea coast. This area is characterized by its socio-economic importance for Egypt. The Eastern Harbour is a comparatively tiny, shallow, and semicircular basin. The Harbor is in the middle of Alexandria’s coastline. The primary metropolitan sewage pumping station is the Quit Bay pumping station, which is the major sewage tube that begins in the center section of Alexandria. Additionally, a variety of human activities, including land-based effluents, yacht sport, fishing, boat industry (boatbuilding and sailing boats), and recreation anchored in the harbor, have a significant impact on it (Abdel Ghani et al., 2013).

The Nile Delta coastal area of Egypt is a delicate and highly dynamic environment subject to various anthropogenic activities such as aquaculture, industrial centers, agricultural land, tourism, ports, and other activities. Also, the Nile Delta’s coastline sector confronts a variety of environmental problems, like erosion, pollution, reduced productivity, and coastal degradation (Elstohey et al., 2023; Mandour et al., 2021). A complex drainage network collects runoff from a variety of municipal, industrial, and agricultural sources in the northern part of the Nile Delta. The drainage network empties treated wastes (partially) into the coastal lagoons and the promontories of the Nile. Ultimately, these wastes are released into the Mediterranean through the coastal lagoon outlets or directly through drains such as the Kitchener and El-Tabia drains. Many researchers have become aware of the trace metal contamination of coastal sediments as a result of the obvious impact of land-based activities on the coastal area around the Nile Delta (Abdallah, 2017; El Nemr et al., 2007; Elstohey et al., 2023; Kaiser et al., 2012; Mandour et al., 2021; Soliman et al., 2015). These researchers reported that there is trace metal pollution in the water and sediments of the coastal area of the Nile Delta, in coastal lagoons, and certain drains. Hotspots for metal pollution have been identified, including the Delta coastal lagoons and Abu Qir Bay to the west of the Nile Delta (Mandour et al., 2021; Shams El-Din et al., 2014).

Finally, the Bardawil lagoon, lagoon is a natural depression on the north coast of the Sinai Peninsula that is separated from the Mediterranean Sea by two artificial openings in the west. Most of the high-quality fish produced at the Bardawil Lagoon is exported to Europe, giving it a significant economic impact and a reputation throughout the world (Said, 2022). These sectors have been chosen and subjected to several point sources of pollution, whether it was agricultural, industrial, domestic water and sewage, or fishing activities.

The management of any environmental system is a continuous and systematic cycle that involves four steps: planning, doing, checking, and acting (Shaaban, 2022). During the planning phase, the primary goals of the strategy are described based on national, regional, and legal restrictions. Then, in the doing phase, the planned processes and pollution control solutions are implemented and operated. The check phase measures the performance of these processes through continuous monitoring, key performance indicator analysis, and evaluation of their effectiveness. Finally, the act or improvement phase identifies errors and defects based on the review stage results, and corrective actions are developed to improve the process (Aboul Dahab & Shaaban, 2017, 2020). Consequently, for an appropriate management strategy for the Egyptian Mediterranean coasts, the complete management cycle should be applied.

The current work focuses on the third phase, the check, or review phase, which involves characterizing the performance indicators of an applied cost management strategy. This includes measuring the degree of sediment quality improvement through (1) monitoring of sediment quality indicator parameters such as metal content, (2) assessing the concentration trend of metals (temporally and spatially), (3) comparing the metal concentrations with national and international guidelines, and (4) converting the concentration values to an easy and understandable description and meaning for decision-making by applying various pollution indices. Finally, the study will suggest more corrective and improvement actions.

The research investigates levels of eight metals (including Fe, Cu, Zn, Mn, Ni, Co, Pb, and Cd) to evaluate sediment pollution, distribution pattern, and potential ecological risks using pollution indices (PI), contamination factor, geoaccumulation factor, enrichment factor, and Pollution Load Index and sediment quality guidelines (SQGs). Improved protection is required for the Mediterranean coastline against the numerous and growing stresses arising from the development of industrial, urban, and tourism sectors, along with other human-induced coastal areas (El-Masry, 2022). Thus, continuous assessment of these sectors should be taken into consideration for the conservation of Mediterranean coasts, not only Egyptian Mediterranean coasts. In addition to assessing the metal content along the delta region perpendicular to the Egyptian Mediterranean Sea coast, this is considered to be the first record for applying pollution indices for evaluating the quality of the sediment and comparing the metal levels in the studied region with others worldwide.

## Materials and methods

### Area of study

**Fig. 1:**
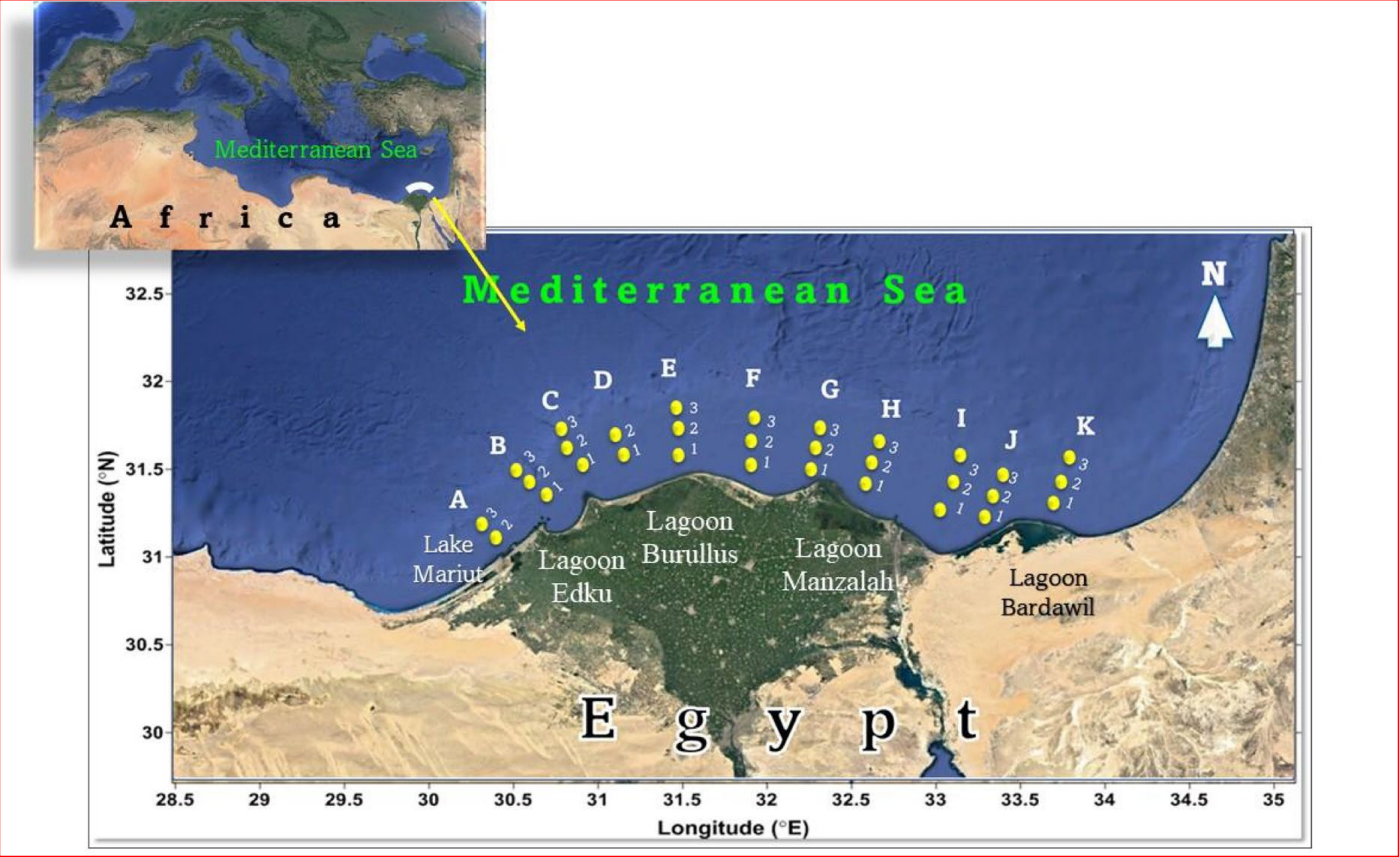
Satellite images showing the location of sampling sites along the investigated area, where **A** = Eastern Harbour, **B** = Abu Qir, **C** = Rosetta, **D** =Abu Khashaba, **E** = Burullus, **F** = Baltim, **G** = Gamasa, **H** = Damietta, **I** = Manzalah, **J** = Bardawil Western Inlet, and **K** = Bardawil eastern inlet sector

Thirty-one surface sediment samples were collected from eleven sectors: Eastern Harbour (A), Abu Qir (B), Rosetta (C), Abu Khashaba (D), Burullus (E), Baltim (F), Gamasa (G), Damietta (H), El Manzalah (I), Bardawil lagoon western inlet (J), and Bardawil lagoon eastern inlet (K) perpendicular to the Egyptian Mediterranean Sea coast (Fig. 1). The study area expanded from west to east, from Alexandria Governorate (at Ras El-Ten) to North Sinai Governorate (at Arish), respectively. The investigated area covered about 350 km and 30 km inside the sea. Each sector was presented by three stations (1, 2, and 3 representing the near, middle, and far shore sediments, respectively). Exceptionally, the Eastern Harbour and Abu Khashaba sectors were subdivided into two stations. The detailed sampling locations are presented in supplementary materials (Table S1).

### Geomorphology of study area

The study area is the inner shelf of the Nile Delta, which was formed in the late Miocene by the Nile Delta’s progressive sedimentation on the Nile Estuary (Mandour et al., 2020). Historically, the Nile River has been the primary source of suspended sediments in the Eastern Mediterranean. The Aswan High Dam and other twentieth-century constructions, such as several dams and barrages built along the Nile River and on its promontories, have prevented the Nile flood from occurring since the Nile was dammed at Aswan (Frihy & Lawrence, 2004). Consequently, the Nile Delta coast’s sediment supply has been drastically reduced, which has had a drastic impact on the region’s geomorphology and caused severe erosion and shoreline retreat. This has prompted ongoing efforts to protect the coast to reduce further degradation of the coastline (Frihy, 2017). The general eastward longshore current that transports sediments from the Nile Delta to the eastern Mediterranean coasts dominates the sediment transport in the inner shelf of the Nile Delta (Mandour et al., 2020).

The sediments of Nile Delta coastal areas have an average grain size ranging from fine to very fine sands. Nearshore sediments are coarser in texture than farshore sediments. The farshore sediment contains a higher silt content than the nearshore sediment (Mandour et al., 2021).

### Sample collection and metal analysis

During June 2021, 31 surface sediment samples were collected using a Van Veen grab sampler. The samples were intended for metal analysis. To prevent contamination from the metallic component, a plastic spoon was used to cut the samples from the grab. Then, samples were freeze-dried in self-sealing polyethylene bags, followed by their grounding with an agate mortar. Three replicates of 0.4–0.5 g of each dried and fine-ground sediment sample were completely digested in Teflon vessels at 85 °C with a mixture of HNO_3_, HF, and HClO_4_ (3:2:1 v/v), for 12 h (Thabet et al., 2023). Metal concentrations were determined using a Shimadzu AA-6800 flame atomic absorption spectrophotometer (AAS). The results were expressed in μg/g dry weight, except iron, which was expressed in mg/g dry weight.

Quality control and quality assurance were considered during sampling, digesting, and analyzing metals contents in samples. Lab wares were rinsed with diluted nitric acid and then by deionized water. All reagents and metal stock solutions were of analytical grade (Merck, Darmstadt, Germany). The working standards were prepared using metal-free distilled water. The method stability and accuracy were measured using reference standard material (SD-M-2/TM, maritime sediments), and each recorded sample reading was the average of two times readings.

### Pollution indices

Several types of indices were applied to evaluate the ecological state of the sediments: contamination factor (CF), degree of contamination (C_deg_), Nemerow Integrated Pollution Index (NIPI), geoaccumulation factor (I_*geo*_), enrichment factor (EF), and Pollution Load Index (PLI) as the pollution indices in addition to Potential Ecological Risk Index (PERI) and formulas expressed in the supplementary material (Table S2).

### Statistical analysis

The distributions of metals in the sediments were visualized using a heat map and evaluated through cluster analysis using Origin Pro 2021 software (OriginLab® Corporation). The relationships between different metals were measured using the Pearson Correlation coefficients with a significance level of *P* ≤ 0.05. The spatial distributions for different pollution indices were illustrated using Surfer 18 software.

## Results and discussion

### Distribution of metals

**Fig. 2:**
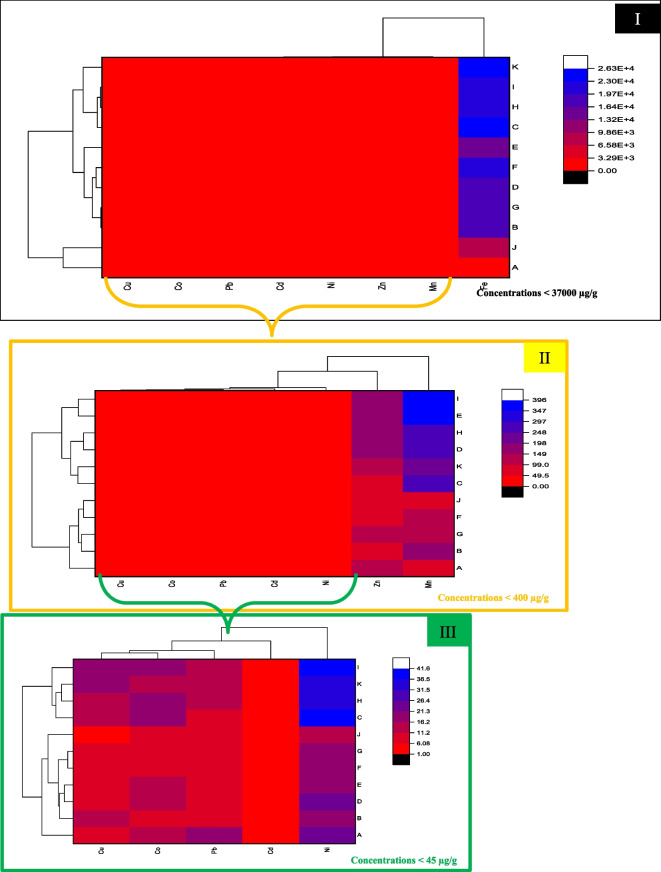
Heat map shows the distribution of mean levels of **I** eight studied metals, **II** seven studied metals, and **III** five studied metals of the study area, indicating their significant variations and interactions. The left tree diagram represents the clustering of areas. The upper tree diagram indicates the clustering of metals with correlation

The abundance of metal concentrations in the current study is illustrated in Fig. 2. The average values of the studied samples are shown in Table 1. Generally, the investigated metals can be sorted in descending order as follows: Fe > Mn > Zn > Ni > Co > Cu > Pb > Cd. The results support previous studies that found that the metals were arranged in similar order and at similar concentrations (dry weight) in most studied locations (Abdel Ghani et al., 2013; El-Amier et al., 2021; El-Metwally et al., 2021; El-Sorogy et al., 2016; Redwan & Elhaddad, 2020, 2022; N. F. Soliman et al., 2015).

**Table 1:** Range and mean of total metals (μg/g) in the study area in addition to the sediment quality guidelines (SQGs) and percentages of samples exceeding SQGs

Metals	Range and mean metal concentrations				Sediment quality guidelines (SQGs)				% of samples exceeding SQGs					
	Min.	Max.	Average	± SD	TEL	PEL	ERL	ERM	< ERL	ERL–ERM	> ERM	< TEL	TEL–PEL	> PEL
Cu	1.2	33.2	11.7	10	19	108	34	270	100	0	0	77	23	0
Zn	3.1	279.9	119.8	81	124	271	150	410	65	35	0	61	39	0
Ni	4.4	68	26.3	18	18	36	30	50	71	13	16	39	35	26
Pb	0.4	25.1	10.2	7	30.2	112	46.7	218	100	0	0	100	0	0
Cd	0.7	4.8	2.1	1	0.7	4.2	1.2	9.6	13	87	0	0	97	3
Co	2	34.7	13.5	9										
Mn	1.9	961.9	213.2	221										
Fe*	1.2	36.7	18.1	11										

*Min.* minimum, *Max.* maximum, *SD* standard deviation; *metal concentration expressed as mg/g

Statistically, it was clear that the distribution of metals can be categorized into three main groups according to their concentrations (Fig. 2). The first group contained extremely high levels of Fe with concentrations < 37,000 μg/g (Fig. 2I). Iron is the most prevalent metal in sediments, where Fe mainly has a lithogenic origin (Aydi et al., 2022). The Fe level varied between minimum levels of about 1300 μg/g (at the middle of both Eastern Harbour and the western inlet of Bardawil sectors) and maximum concentrations of about 36,000 μg/g (at the nearshore sediments of Rosetta and Manzalah sectors). Extremely high Fe concentrations were generally associated with the presence of Fe in the Earth’s crust, where it was classified as the tenth most abundant element and made up roughly 34.6% of the crust’s total mass (Shaaban et al., 2022).

The second group (Fig. 2II) contained intermediate concentrations of Mn and Zn < 400 μg/g. Manganese distribution was like that of Fe. Concentrations of Mn fluctuated from 1.9 (at the far shore sediments of the Baltim sector) to 961.9 μg/g (at the near-shore sediments of Manzalah). Similarly, Zn contents ranged between 3.1 and 279.9 μg/g. The Zn and Mn concentrations primarily controlled natural factors, particularly the Nile water supply, coastal erosion, and deposition (Badawi & Magdy, 2023). Results revealed that Zn concentrations were also affected by industrial sources (agricultural activities and industrial wastewater), as reflected in the relatively high values in Manzalah and Eastern Harbour. Moreover, the nearshore sediments in these two sectors were enriched by Zn content comparable to offshore (> three times). These results agreed with other research that recorded high concentrations of Zn affected mainly by anthropogenic activities in coastal regions, especially industrial effluent discharge (Achary et al., 2016; Boxall et al., 2000; Christophoridis et al., 2019; Padhi et al., 2013; Sungur et al., 2020).

The third group (Fig. 2III) involves comparatively low levels (with concentrations of < 40 μg/g) of Cu, Co, Ni, Pb, and Cd [Cu 1.22 (Baltim)–33.2 (Manzalah), Co 2.01 (Baltim)–34.72 (Manzalah), Pb 0.4 (Rosetta)–25.1 (Eastern Harbour), Cd 0.7 (Burullus)–4.8 (Eastern Harbour), and Ni 4.4 (Baltim)–68 (Manzalah)]. These groups include non-essential and toxic metals as previously noticed by (Dökmeci et al., 2019; Raza’i et al., 2022) in marine sediments of the Marmara Sea along Tekirdağ coast and Bintan Island, Indonesia, respectively.

The average concentrations of the studied metals were below and close to those corresponding values in the continental crust and shale values, excluding Zn and Cd. The metal industry, used phosphate fertilizers, cremated solid waste, power plants, toxic waste from industrial plants, and sewers were responsible for Cd emissions into the aquatic environment (Tepe et al., 2022). Moreover, a combination of industrial, agricultural, and municipal wastewater in the drains appears to be the source of the observed high Zn levels (El-Metwally et al., 2021). Generally, the presence of metals in the delta coastal region is caused by both the anthropogenic addition of point sources and the natural geochemical provenance of the sediments themselves (Mandour et al., 2021).

Regionally, the Manzalah sector attained the most enriched sediments, while the Baltim sector showed the lowest metal levels. This may be because of the impact of Manzalah Lagoon that was shown high levels of pollution over the past six decades because of many point sources, including sewage, agriculture, and industrial effluents, particularly from the southern and western boundaries. Also, six large, very contaminated drains provide water for the lagoon’s drainage system, producing a hot spot of metal pollution (Redwan & Elhaddad, 2022).

On the other hand, cluster analysis was used to identify the similarity groups among the sampling sites and groups of metals based on metal concentrations (Ustaoğlu & Tepe, 2019). Spatially and based on the cluster analysis (Fig. 2), the studied area was classified into two main groups, with 100% dissimilarity. The first group includes the Eastern Harbour and the western inlet of Bardawil Lagoon sectors (A and J); this may be due to both regions being relatively shallow and having small openings with the Mediterranean Sea for water exchange. Moreover, both sectors had no direct discharge from agricultural drainage water, and Eastern Harbour was the only sector without industrial activities (Abdel Ghani et al., 2013). The other group was subdivided into two subgroups, the first subgroup included Abu Qir, Gamasa, Abu Khashaba, Baltim, and Burullus, and the second subgroup involved Rosetta, Damietta, El Manzalah, and Bardawil Lagoon eastern inlet.

**Fig. 3:**
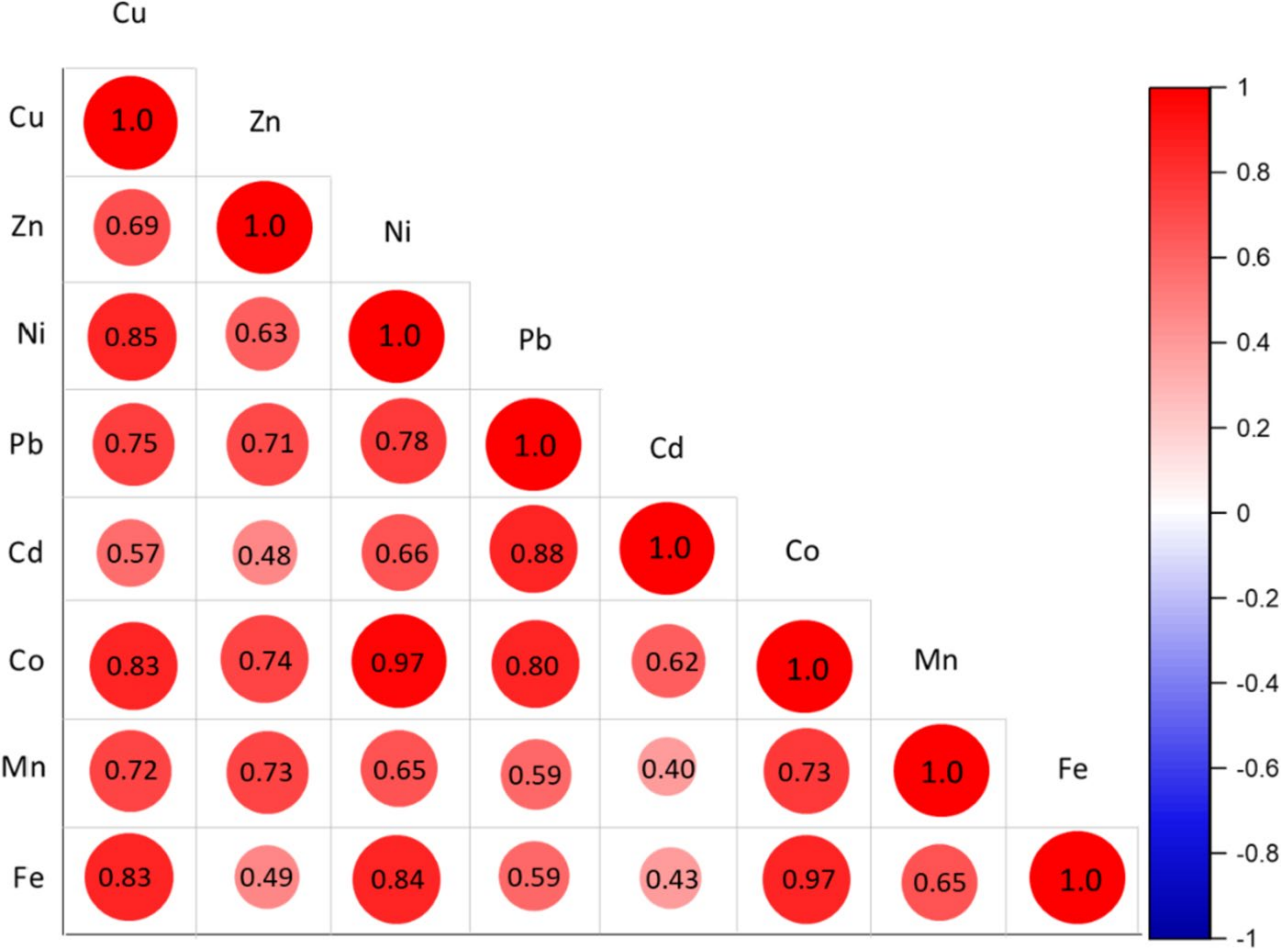
Correlation coefficient matrix

The statistical correlation analysis shows that all metals in the surface sediments of the studied region are positively correlated with each other (*r* > 0.40, *P* ≤ 0.05) (Fig. 3). The figure revealed a highly significant correlation between Cu, Ni, Co, and Fe (with r of about 0.80) indicating their common source of origin, as confirmed by (Redwan & Elhaddad, 2022; Thabet et al., 2023). At the same time, Zn and Cd showed a less positive correlation with Fe (*r* < 0.50) which coincided with the different sources of metals. The Cd and Zn mainly originate from anthropogenic activities, where the studied region was subjected to several point sources of pollution such as agricultural, industrial, domestic water, sewage, or fishing activities, as mentioned before.

The comparison between the mean metals’ levels of the current study and other surface sediments in the Mediterranean coastal area and worldwide is presented in Table 2. In the present study, the levels of delta Mediterranean coastal sediments were slightly higher than those corresponding levels of previous work on the same region (Mandour et al., 2021). Metal levels remained much higher compared to Algerian Mediterranean Sea sediments and Khnifiss Lagoon, Mediterranean Sea, Morocco (Aydi et al., 2022; Tnoumi et al., 2020). Whether, in comparison to other studies in the Mediterranean coastal waters, concentrations of Cu, Ni, Pb, Co, Fe, and Mn are lower than those evaluated in the Coast of Aliağa, the Mediterranean Sea in Turkey, Bay of Quiberon, and Gulf of Morbihan of South Brittany waters, France. In comparison to Red Sea, the present study’s metal levels are much higher than those in the Red Sea (except Mn results because of mining activities in the area) (Al-Kahtany, Nour, El-Sorogy, & Alharbi, 2023). Compared to similar studies worldwide, metal concentrations in the present remained lower than those of estuarine sediments in the Black Sea (Aydın et al., 2023). Cu and Pb are lower concentrations than those of Pearl Bay in China (Yang et al., 2022). Ni, Fe, and Mn are lower than Dhamara, coast estuary India (Sahoo & Swain, 2023). In comparison with previous studies in the Mediterranean coastal waters and worldwide, the sediments of the present study are enriched with higher concentrations of Cd and Zn.

**Table 2:** Comparison table of metal concentrations (μg/g) in other Mediterranean Sea coastal sediments and worldwide

	Cu	Zn (μg/g)	Ni (μg/g)	Pb (μg/g)	Cd (μg/g)	Co (μg/g)	Fe (μg/g)	Mn (μg/g)	References
Delta region coastal sediments, Egypt	11.7 1.2–33.2	119.8 33.2–179.9	26.3 4.4–68	10.2 0.4–25.1	2.1 0.7–4.8	13.5 2–34.7	18,000.1 1200–36,700	213.2 1.9–961.9	Present study
Dhamara coast estuary, India	7.4–30.5	47.5–93.9	45.4–90.7	5.9–19	0.7–1.3	-	28,508.9–57,405.9	430.6–820.9	(Sahoo & Swain, 2023)
Estuarine sediments, Black Sea	82.7	155	45	93.7	1	18	56,659.8	1168.5	(Aydın et al., 2023)
Red Sea	0.02–1.56	0.99–19.56	0.06–4.83	0.41–5.46	0.01–0.19	0.12–2.14	2252–5292	141–2104	(Al-Kahtany, Nour, Giacobbe, et al., 2023)
Bizerte coastal line, Tunisia	0.01–5.1	0.8–6.3	0.2–1.6	0.3–41.5	0.01–0.2	0.02–0.3	113,000–955,000	0.8–7.4	(Aydi et al., 2022)
Algerian Mediterranean Sea sediments, Algeria	1.1–10.4	5.3–45.7	0.8–54.9	1.3–11.5	0.1–2.3	-	-	-	(Aydi et al., 2022)
Pearl Bay, China	0.3–67.9	6.4–110.5	-	9.6–68.7	0.02–2.7	-	-	-	(Yang et al., 2022)
Delta coastal sediments, Egypt	-	16.8–140.6	8.4–66.3	2.8–19.6	0.04–0.4	5.2–33.7	-	-	(Mandour et al., 2021)
Bay of Quiberon, South Brittany waters, France	2.6–40.9	4.6–98.9	-	3.6–28	0.01–0.2	-	-	-	(Ong et al., 2021)
Gulf of Morbihan, South Brittany waters, France	1.2–48.6	4.8–89.9	-	2.4–42.7	0.01–0.3	-	-	-	(Ong et al., 2021)
Khnifiss Lagoon, Mediterranean Sea Morocco	2–12.6	15–101	10–26.1	2.5–12.4	0.1–0.4	1.2–7.1	-	-	(Tnoumi et al., 2020)
The coast of Aliağa, Mediterranean Sea Turkey	20–703	86.8–129.0	28–240	91.3–751	0.06–3.9	-	-	283–1192	(Neşer et al., 2012)
Earth crust background	45	95	68	20	0.3	19	47,000	850	(Turekian & Wedepohl, 1961)

### Pollution and ecological risk status

**Fig. 4:**
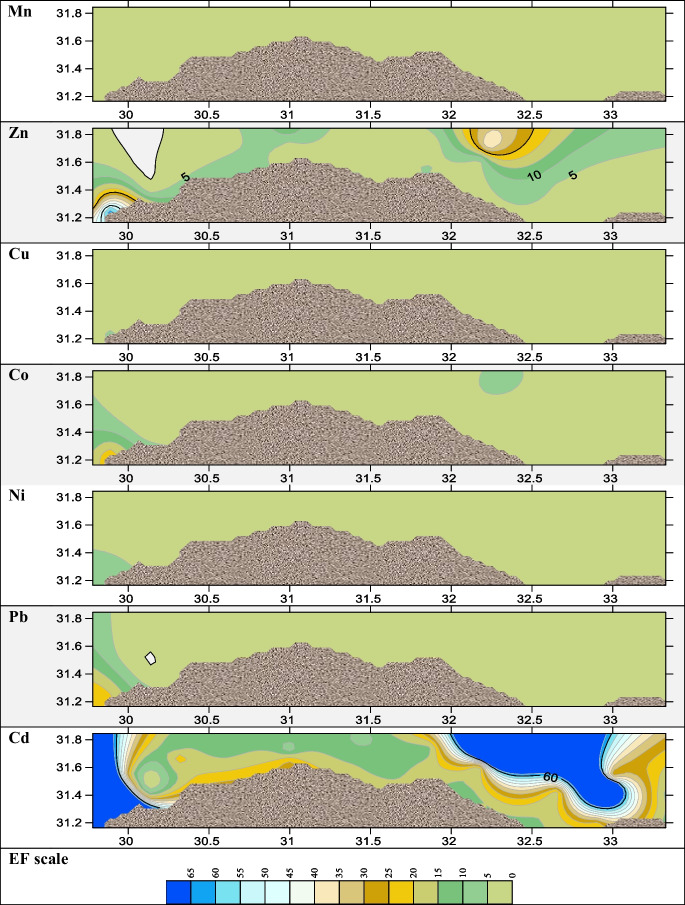
The spatial distributions of the enrichment factor (EF)

**Fig. 5:**
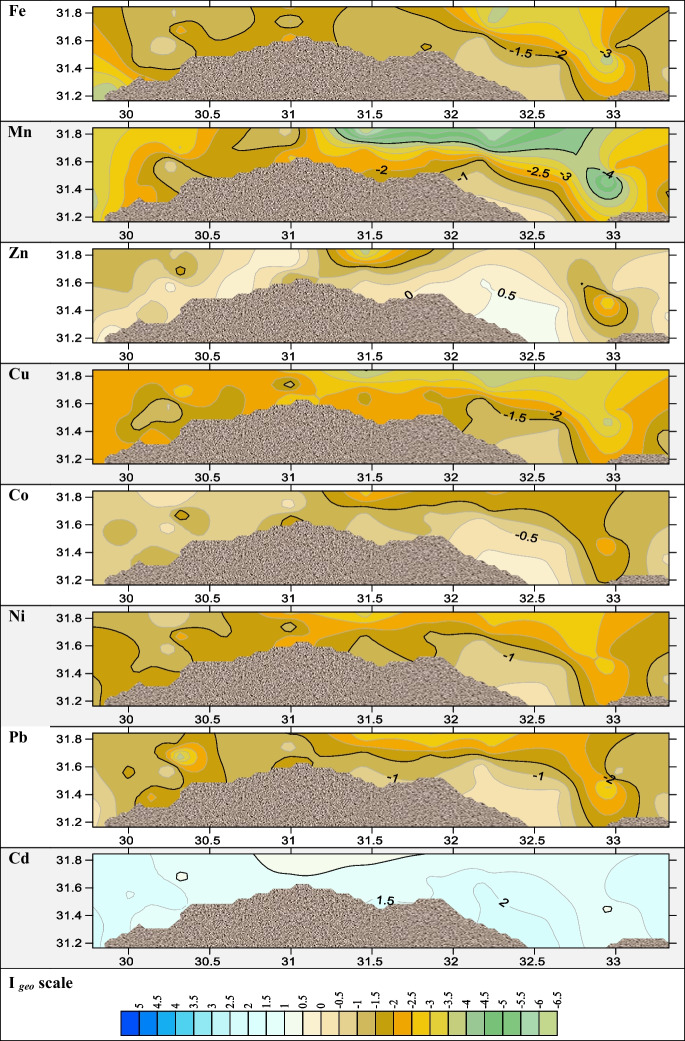
The spatial distributions of geoaccumulation factor (*I*_geo_)

**Fig. 6:**
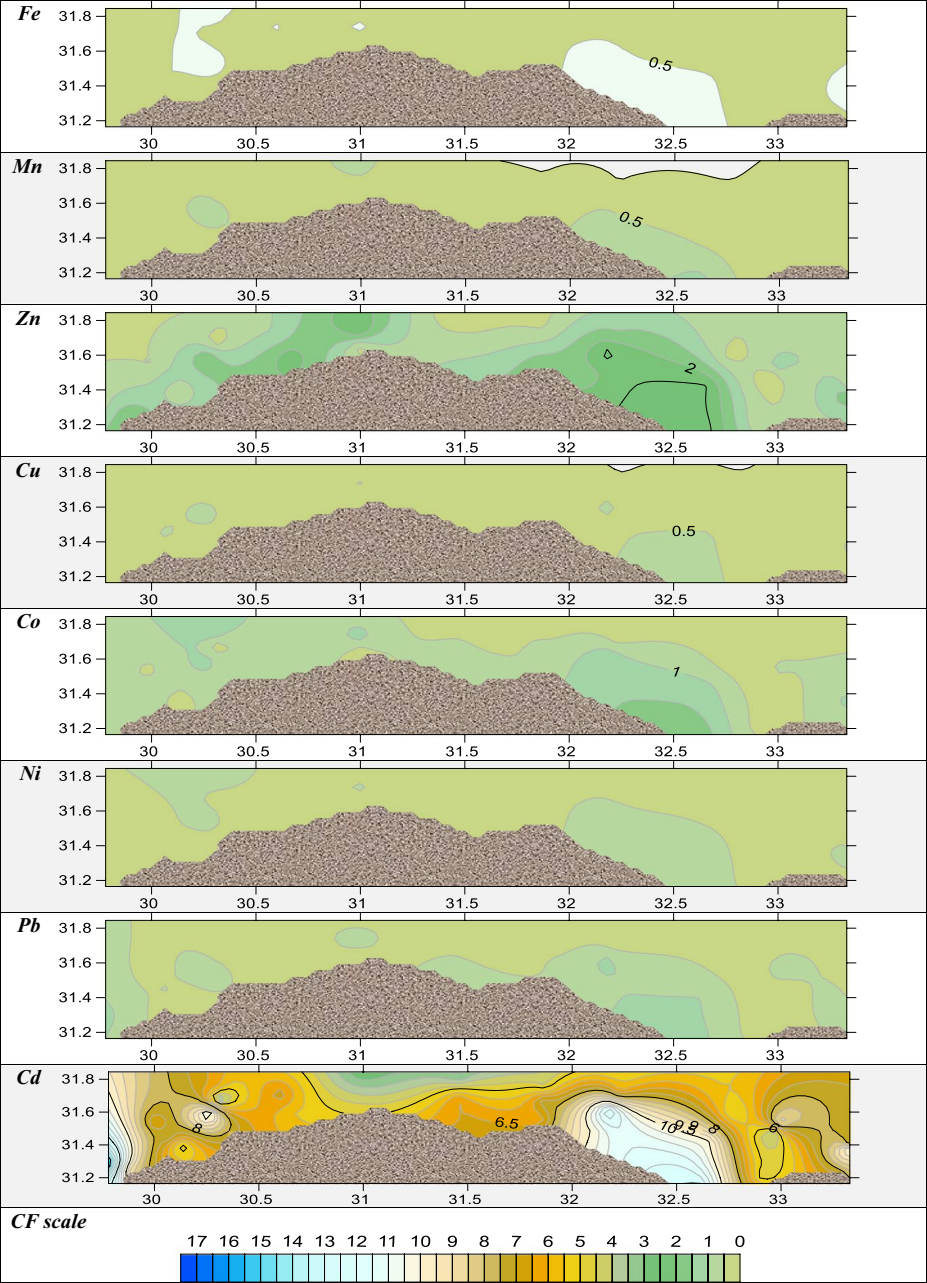
The spatial distributions of contamination factor

For assessing the pollution level of metals in sediments or soils, various pollution indices are used. These indices are classified as either single or integrated, depending on whether one or more elements are being assessed. (Ferreira et al., 2022). Several single approaches were applied in the present study to appraise the pollution indices, such as EF, *I*_geo_, and CF. EF was used to compare various contamination sources, whether they were caused by anthropogenic or natural causes, and to evaluate the degree of sediment metal pollution (Kadhum, 2020). The results of EF (Fig. 4) revealed that most of the investigated metals, Co, Cu, Ni, Pb, Mn, and Zn, were depleted to mineral levels (EF values < 2) except the Eastern Harbour in Co, Cu, Ni, Pb, and Zn with extremely high EF which indicates severe enrichment of sediment metal pollution (Kadhum, 2020); from Fig. 4, it was indicated that cadmium results at most locations with high enrichment. *I*_geo_ is applied to evaluate the metal contamination degree (Astatkie et al., 2021). According to calculated *I*_geo_, most metals Co, Cu, Ni, Pb, Mn, and Zn fell into a class of < 0 of geoaccumulation index with an unpolluted indication as shown in Fig. 5. *I*_geo_ values of Cd lay between classes 1 and 2 which categorized from unpolluted to a moderately polluted environment. The contamination factor of all sampling locations was low contaminated with metals Fe, Mn, Cu, Co, Ni, and Pb < 1 as illustrated in Fig. 6, while it was moderately contaminated with Zn at Eastern Harbour, Rosetta, Abu Khashaba, Burullus, Damietta, and El Manzalah. The CF of Cd in sediments of all the studied regions were highly contaminated as revealed in Fig. 6. The order of total mean contamination factor of metals in the sampling locations was decreased in order Cd > Zn > Co > Pb > Ni, Fe > Mn, Cu as 7.1, 1.3, 0.7, 0.5, 0.4, 0.4, 0.3, and 0.3, respectively meaning that the study area is heavily polluted by Cd varied from moderately to low contaminated with other metals, which indicated that the high degree of anthropogenic pollution and metal contamination from a point source.

**Fig. 7:**
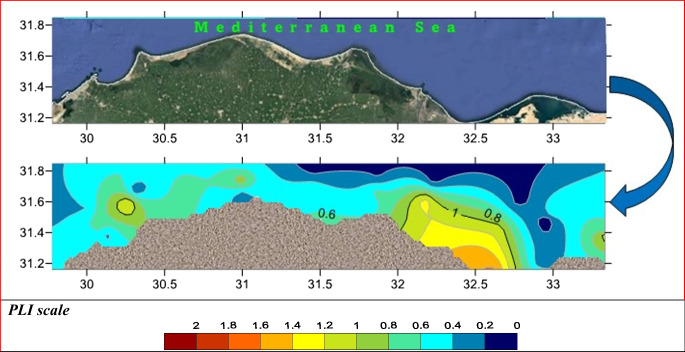
The overall spatial distributions of pollution loading index

**Fig. 8:**
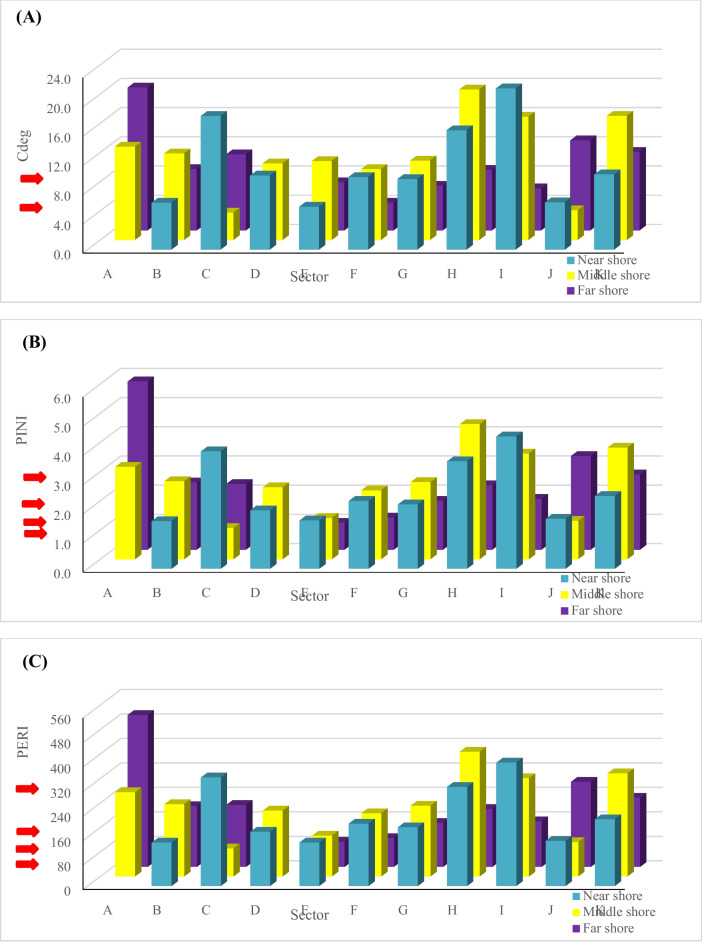
The spatial distributions of the degree of contamination, *C*_deg_ (**A**), Nemerow Integrated Pollution Index (NIPI) (**B**), and the Potential Ecological Risk Index (PERI) (**C**), respectively

According to integrated pollution indices, PLI, C_deg_, NIPI, and PERI were implemented. The PLI was used to evaluate the extent of metal contamination of sediments in various locations (Soliman et al., 2022). The overall and integrated index of PLI illustrated that Eastern Harbour, Abu Qir, Abu Khashaba, Burullus, Baltim, Gamasa, and Bardawil Lake inlet west are below and at the baseline levels. Meanwhile, the results revealed that Damietta, EL Manzalah, Rosetta, and Bardawil lake inlet east are polluted areas, as presented in Fig. 7. The *C*_deg_ was applied to determine the degree of contamination (Nour et al., 2022). According to Fig. 8A, *C*_deg_ varied from 6 to 24 which revealed that the area of study lay between medium to highly contaminated regions where Eastern Harbour, Damietta, and EL Manzalah were recorded as highly contaminated regions. At the same time, NIPI was utilized to assess the degree of pollution restored by heavy metals (Chai et al., 2021). According to NIPI, most of the present study area lies in a moderately level of pollution except Eastern Harbour, Damietta, and Bardawil eastern inlet sector indicating a high level of pollution (> 3) as shown in Fig. 8B. PERI is primarily used to evaluate the harmful effects of heavy metals (HMs) on humans and ecosystems, as well as the toxicity and ecological vulnerability associated with HMs. In many contaminated regions, it also governs our understanding of biological populations (Kumar et al., 2022). The PERI results revealed that Eastern Harbour, Damietta, Manzalah, and Bardawil eastern inlet sectors are highly polluted regions and all other regions lie in considerable pollution levels.

Overall, Eastern Harbour, Damietta, Manzalah, and Bardawil eastern inlet are the most polluted sectors in the investigated regions while the Baltim sector showed the lowest metal levels. This may be due to numerous point sources of pollution that have been present in these regions. Eastern Harbour suffers from sewage pollution and several kinds of human activities including land-based effluents and fishing (enriched with Pb due to using antifouling paints) (Zaghloul et al., 2020). Also, Damietta was impacted by several pollution sources, including industrial (Talkha fertilizer factory and thermal pollution from cooling the condensers of Kafr Saad Electrical station), municipal (El-Serw City), and agricultural drains (Redwan & Elhaddad, 2020). Finally, Manzalah Lagoon and Bardawil (eastern inlet) impacted by sewage, agriculture, and industrial effluents, which creates hotspots for metal pollution (Ahmed & Mohammed-AbdAllah, 2023; Shalloof et al., 2023).

To assess the negative biological consequences and sediment toxicity, various sediment quality guidelines have been developed. Two sets of sediment quality guidelines (SQGs) were used to assess the ecological risks and potential toxicity of the sediments to aquatic ecosystems (TEL–PEL and ERL–ERM). Whereas the PEL and ERM reveal a frequent harmful biological effect, low values of the TEL and ERL suggest a minimally adverse biological impact (Tepe et al., 2022). Evaluation of SQG comparisons and probable effects on metal content were represented in Table 1. Cu, Zn, Ni, Pb, and Cd levels in sediment samples are compared to TEL-PEL SQGs, and the results show percentages below TEL values of 77, 61, 39,100, and 0%, respectively. Contrarily, the values of these metals in the sediment samples are 23, 39, 35, 0, and 97% falling within the range between the TEL and PEL, while the values in the remaining samples are greater than PEL. The abundance of Cu, Zn, Ni, Pb, and Cd concentrations in sediment samples, however, is below the ERL values, with contributions of 100, 65, 71, 100, and 13%, respectively, when compared to the ERL-ERM SQGs. In terms of Cu, Zn, Ni, Pb, and Cd, 0, 35, 13, 0, and 87% of all sediment samples, respectively, lie between ERL and ERM levels.

## Conclusion

The current study provides an overview of the investigated metal levels and distribution in surface coastal sediments from the delta region perpendicular to the Egyptian Mediterranean Sea coast. According to the mean concentrations of metals, the investigated metals can be sorted as follows in descending order: Fe (18.1 mg/g) > Mn (213.2 μg/g) > Zn (119.8 μg/g) > Ni (26.3 μg/g) > Co (13.4 μg/g) > Cu (11.7) > Pb (10.2 μg/g) > Cd (2.1 μg/g). Most metals, except for Zn and Cd, were below the background values of the earth’s crust. According to anthropogenic activities, commercial, industrial, and agricultural activities are the main sources of metals. However, metal levels were also evaluated using ecological indices, which are not only used as indicators of sediment quality but are also correlated with results from different sources. The EF values indicated that most metals were depleted to mineral levels. While the *I*_geo_ revealed that the surface sediments were moderately polluted with Cd and unpolluted with other metals, the CF values reflected that the sediments were highly polluted with Cd and lowly contaminated with other metals.

Overall, regionally according to integrated pollution indices, Eastern Harbour, Damietta, Manzalah, and Bardawil eastern inlet are the most polluted sectors in the current study, while the Baltim sector showed the lowest metal levels.

However, the current study covers the gap and gives a description of the distribution of selected essential and other toxic metals and their role in measuring sediment quality, pollution status, and ecological significance at the southeastern part of the Mediterranean coastal line along one-third of the Egyptian coast. More details about the biogeochemical cycles and speciation of Zn and Cd not only in the sediments but also in the water column, particularly in the hot spots, are recommended as a way of managing and controlling pollution. More focus on a wide range of non-essential toxic metals such as mercury (Hg), arsenic (As), and chromium (Cr) should be paid.

On the other hand, the development of the current complicated coastal drainage system because of runoff from various point sources of pollution in the northern sector of the Nile Delta (municipal, industrial, and agricultural) is one of the most significant issues along the delta Egyptian Mediterranean coasts. The new coastal drainage system is helping to protect and improve fish stocks and enhance water conservation.

### Supplementary information

ESM 1 (DOCX 53 kb)

## Data Availability

The datasets generated during the current study are available from the corresponding author on request.
