# Supplementary material for: Ecological risk assessment of metal pollution in the surface sediments of delta region, Egypt
Source: Environ Monit Assess. 2024 Mar 11;196(4):351. doi: 10.1007/s10661-024-12481-w (PMC10925576; doi:10.1007/s10661-024-12481-w)
Supplement: Supplementary file 1 — (DOCX 53 kb) [file 10661_2024_12481_MOESM1_ESM.docx]

# Supplementary Materials

**Table S1:** Sample locations of sediments collected from the Mediterranean Sea coast.

| **Station** | **Sector** | **Long. (^o^E)** | **Lat. (^o^N)** | **Station** | **Sector** | **Long. (^o^E)** | **Lat. (^o^N)** |
| --- | --- | --- | --- | --- | --- | --- | --- |
| **A2** | Eastern Harbour | 29.881 | 31.248 | **G1** | Gamasa | 31.842 | 31.562 |
| **A3** |  | 29.78 | 31.291 | **G2** |  | 31.861 | 31.678 |
| **B1** | Abu Qir | 30.129 | 31.399 | **G3** |  | 31.873 | 31.752 |
| **B2** |  | 30.075 | 31.45 | **H1** | Damietta | 32.109 | 31.488 |
| **B3** |  | 30.005 | 31.543 | **H2** |  | 32.175 | 31.616 |
| **C1** | Rosetta | 30.264 | 31.593 | **H3** |  | 32.23 | 31.721 |
| **C2** |  | 30.311 | 31.678 | **I1** | Manzalah | 32.62 | 31.165 |
| **C3** |  | 30.299 | 31.756 | **I2** |  | 32.66 | 31.43 |
| **D1** | Abu Khashaba | 30.656 | 31.593 | **I3** |  | 32.767 | 31.595 |
| **D2** |  | 30.602 | 31.713 | **J1** | Bardawil western inlet | 32.904 | 31.208 |
| **E1** | Burullus | 30.997 | 31.647 | **J2** |  | 32.963 | 31.479 |
| **E2** |  | 30.993 | 31.736 | **J3** |  | 32.989 | 31.532 |
| **E3** |  | 30.989 | 31.845 | **K1** | Bardawil eastern inlet | 33.27 | 31.226 |
| **F1** | Baltim | 31.431 | 31.547 | **K2** |  | 33.322 | 31.38 |
| **F2** |  | 31.451 | 31.686 | **K3** |  | 33.329 | 31.404 |
| **F3** |  | 31.454 | 31.814 |  |  |  |  |

Several types of indices were applied to evaluate the ecological state of the sediments; Contamination factor (CF), Geoaccumulation factor (I*_geo_*), Enrichment factor (EF), and Pollution Load Index (PLI).

**Contamination Factor:**

The contamination factor is an effective tool for monitoring pollution over a period and evaluating the pollution of environmental single substances. ***Turekian and Wedepohl (1961)*** The contamination degree is generally expressed as CF (Astatkie et al. 2021) as shown in equation**,**

$\boldsymbol{Cf=}\frac{\boldsymbol{C metal}}{\boldsymbol{C backgound value}}$ **,**

Where ***C_metal_*** is the total metal concentration and ***C_Background_*** the average background value of the element from a geologically similar and uncontaminated area. ***Turekian and Wedepohl (1961)*** reported the geochemical background values of the metals used in the shale. The CF value may correspond to one of the levels of contamination, where CF varied as; < 1 (low polluted); 1 ≤ CF ≥3 moderately polluted; 3 ≤ CF ≥6 considerably polluted; and CF > 6 (very polluted).

**Degree of contamination (C _deg._):**

C _deg._ =∑ 𝐶𝐹,

where CF is the contamination factor. Sediments are categorized into low, medium, and high contamination according to the C _deg._ values of < 4, 4-8, and > 8, respectively, (Nour et al. 2022).

**Nemerow Integrated Pollution Index (NIPI):**

$NIPI= \bar{\sqrt{\frac{P_{max}^{2}+p_{mean}^{2}}{2}}}$,

Pi = $\frac{Ci}{Si}$

Where Ci is the exact metal i concentration, Si is the background value of metal I, P_max_ is the maximum value of sediment metal pollutant index, and P _mean_ is the average value. The NIPI values are classified into 5 groups, namely, no pollution (≤ 0.7), warning line of pollution (0.7 - ≤ 1), low level of pollution (1 - ≤ 2), moderate level of pollution (2 - ≤ 3), and high level of pollution (> 3), (Chai et al. 2021).

**Geoaccumulation factor:**

This method assesses the degree of metal pollution in terms of enrichment classes based on increasing the numerical values of Igeo. (Astatkie et al. 2021) applied the formula of

***Igeo*** of ***I_geo_* = Log 2** (**Cn /1.5 Bn**)

where ***Cn*** and ***Bn*** are the measured concentration of metal in sediment and geochemical background value in average shale (***Turekian and Wedepohl (1961)***, respectively. n, 1.5 is the background matrix correction in factor due to lithogenic effects. The ***Igeo*** consists of six grades ranging as follows (0) unpolluted; (1) unpolluted to moderate; (2) moderately polluted; (3) moderate to high polluted; (4) highly polluted and (5) very highly polluted.

**Enrichment factor**

Calculating enrichment factor (EF) is an essential part of geochemical studies in distinguishing heavy metals that originating from natural weathering (***Praveena et al., 2010***). Such technique was greatly applied by normalizing a metal concentration to the texture or compositional characteristics of sediments. Fe was used as a geochemical normalization element to alleviate the variations produced by heterogeneous sediments. **Zhuang and Gao, 2014** applied the formula of

***EF* =** $\frac{(\boldsymbol{M /Fe})\boldsymbol{Sample}}{(\boldsymbol{M /Fe})\boldsymbol{crust}}$

Where, ***(M/Fe)*** sample is the ratio of the metal and Fe concentration of the sample, and ***(M/Fe)*** crust is the ratio of the metal and Fe in the crust. Fe was used as a geochemical normalization element to alleviate the variations produced by heterogeneous sediments. EF values classified as less than 2 (unpolluted), 2 ≤ EF <5 moderate, 5 ≤ EF < 20 significant, 20 ≤ EF < 40 very high and EF more than 40 very polluted.

**Pollution Load Index**

Astatkie et al., 2021; Zhuang and Gao, 2014 stated the formula equation of PLI as

***PLI* =** $\sqrt[\boldsymbol{n}]{\boldsymbol{( CF}\boldsymbol{1}\boldsymbol{x CF}\boldsymbol{2 x CF}\boldsymbol{3 x\ldots\ldots\ldots\ldots x CFn}}$

Where ***n*** is the number of metals and ***CF*** is the contamination factor. PLI divided into three categories from perfection 0, to below 1 which is baseline and more than 1 is polluted.

**Potential Ecological Risk Index (PERI)**

PERI = $\sum_{i}^{n} ( Trf\times CF)$

Trf is the toxic response factor for metals and the risk according to PERI can be grouped into five classes as PERI < 40 (no~low), 40 ≤ PERI < 80 (moderate), 80 ≤ PERI <160 (considerable), 160 ≤ PERI ˂ 320 (high), and PERI ≥ 320 (very high), (Kumar et al. 2022).

**Refernces**

Astatkie, H., Ambelu, A., Mengistie, E., 2021. Contamination of Stream Sediment With Heavy Metals in the Awetu Watershed of Southwestern Ethiopia. Front Earth Sci (Lausanne) 9. <https://doi.org/10.3389/feart.2021.658737>.

Praveena, S.M., Aris, A.Z., Radojevic, M., 2010. Heavy metals dynamics and source in intertidal mangrove sediment of Sabah Borneo Island. Environment Asia 3, 79–83.

Turekian, K.K., Wedepohl, K.H., 1961. Distribution of the Elements in Some Major Units of the Earth’s Crust. GSA Bulletin 72, 175–192. [https://doi.org/10.1130/0016-7606(1961)72[175:DOTEIS]2.0.CO;2](https://doi.org/10.1130/0016-7606(1961)72%5b175:DOTEIS%5d2.0.CO;2).

Zhuang, W., Gao, X., 2014. Integrated Assessment of Heavy Metal Pollution in the Surface Sediments of the Laizhou Bay and the Coastal Waters of the Zhangzi Island, China: Comparison among Typical Marine Sediment Quality Indices. PLoS One 9, e94145. <https://doi.org/10.1371/journal.pone.0094145>.

Chai, L., Wang, Y., Wang, X., Ma, L., Cheng, Z., & Su, L. (2021). Pollution characteristics, spatial distributions, and source apportionment of heavy metals in cultivated soil in Lanzhou, China. *Ecological Indicators*, *125*. https://doi.org/10.1016/j.ecolind.2021.107507

Kumar, V., Pandita, S., & Setia, R. (2022, March 1). A meta-analysis of potential ecological risk evaluation of heavy metals in sediments and soils. *Gondwana Research*. Elsevier Inc. https://doi.org/10.1016/j.gr.2021.10.028

Nour, H. E., Alshehri, F., Sahour, H., & El-Sorogy, A. S. (2022). Evaluation of sediment and water quality of Ismailia Canal for heavy metal contamination, Eastern Nile Delta, Egypt. *Regional Studies in Marine Science*, *56*. https://doi.org/10.1016/j.rsma.2022.102714
